# Expression of COX-2 and bcl-2 in oral lichen planus lesions and lichenoid reactions

**DOI:** 10.3332/ecancer.2014.411

**Published:** 2014-03-20

**Authors:** Alven J Arreaza, Helen Rivera, María Correnti

**Affiliations:** 1 Dental Therapeutics, School of Dentistry, Central University of Venezuela, Caracas 1051, Venezuela; 2 `Raúl Vincentelli’ Oral Pathology Laboratory, Institute of Dental Research, School of Dentistry, Central University of Venezuela, Caracas 1051, Venezuela; 3 `Raúl Vincentelli’ Institute of Dental Research, School of Dentistry, Central University of Venezuela, Caracas 1051, Venezuela

**Keywords:** oral lichen planus, lichenoid reaction, bcl-2, COX-2

## Abstract

Oral lichen planus and lichenoid reactions are autoimmune type inflammatory conditions of the oral mucosa with similar clinical and histological characteristics. Recent data suggest that oral lichenoid reactions (OLR) present a greater percentage of malignant transformation than oral lichen planus (OLP).

**Objective:**

To compare the expression of bcl-2 and COX-2 in OLP and OLR.

**Methods:**

The study population consisted of 65 cases; 34 cases diagnosed as OLR and 31 as OLP. A retrospective study was done, and bcl-2 and COX-2 expression was semiquantitatively analysed.

**Results:**

Fifty-three per cent (18/34) of the ORL samples tested positive for COX-2, whereas in the OLP group, 81% of the samples (25/31) immunostained positive for COX-2. The Fisher’s exact test for the expression of COX-2 revealed that there are significant differences between the two groups, *P* = 0.035. With respect to the expression of the bcl-2 protein, 76% (26/34) of the samples were positive in OLR, while 97% (30/31) were positive in the group with OLP. The Fisher’s exact test for the expression of bcl-2 revealed that there are significant statistical differences between the two groups, *P* = 0.028.

**Conclusions:**

The expression of bcl-2 and COX-2 was more commonly expressed in OLP when compared with OLR.

## Introduction

Oral lichen planus (OLP) is a relatively common mucocutaneous entity. It has a variety of clinical presentations: papular–reticular lesions, linear plaques, erosions, ulcers, and less frequently, hyperchromic or hypochromic macules, or a combination. The prevalence of this condition is 1–4% of the world population with no apparent racial predisposition and a predilection for females [1, 2].

Moreover, oral lichenoid reactions (OLR) is a term used to describe clinical and histopathological lesions similar to OLP but with an identifiable etiologic factor. OLR tend to be unilateral and erosive, with a slightly more diffuse lymphocytic infiltrate with increased eosinophils, plasmocytes, and cytoid bodies than OLP, although it is common to find similar histological characteristics in both entities [1–3].

The malignant potentials for OLP and OLR are still controversial. Recent data suggest that OLR present a greater percentage of malignant transformation than OLP and, although the association between cancer and OLP has been documented in scientific reports, there is no association between squamous cell carcinoma and cutaneous lichen planus [4–6].

Cyclooxygenase 2 (COX-2), which is the inducible form of this enzyme, plays an important role in inflammation and carcinogenesis. Overexpression of COX-2 is important in tumour growth, invasion and metastasis, angiogenesis, and inhibition of apoptosis, all important steps in the development of cancer. The increased expression of COX-2 has been reported in pre-cancerous lesions, in several forms of cancer, including squamous cell carcinoma and in OLP [7].

The B-cell lymphoma-2 (bcl-2) protein was the first protein that was associated with translocations identified in follicular lymphoma. The bcl-2 protein is located in the inner mitochondrial membrane and, to a lesser extent, in the nuclear membrane and the endoplasmic reticulum and plays a fundamental role in cellular protection against apoptosis. The growth of keratinocytes is regulated by a delicate balance between molecules, such as bcl-2 that control cell survival and cell molecules, such as p53 that control and cell death. Bcl-2 is an anti-apoptotic molecule that is inversely related to p53, since its expression prevents cellular apoptosis, modulating the release of cytochrome c by the mitochondria [8, 9].

The importance of the bcl-2 protein in cell death and apoptosis in various oral diseases, such as squamous cell carcinoma, leukoplakia, and other epithelial dysplasias, such as OLP has been a subject of study [10].

Reports suggest that OLP and/or OLR are potentially malignant, and both lesions may be involved in the maintenance of chronic inflammation, a condition that can lead to the transformation of OLP and OLR into squamous cell carcinoma [11, 12]. The expression of bcl-2 in OLP and OLR has not been fully established due to conflicting results [13].

The objective of the present study is to compare the expression of bcl-2 and COX-2 in OLP and OLR lesions.

## Methods

The study population consisted of 65 cases, 34 diagnosed with OLR, and 31 cases of OLP from the Oral Pathology Laboratory, School of Dentistry, Central University of Venezuela (UCV) during 2003–2012. All cases were histologically classified, according to the Van der Waal and Van Meij criteria, in 2003 [14].

Three-micrometer sections on blocks were done, deparaffinized, and antigen retrieval was performed using a commercially available solution (DAKO, Carpinteria, CA, USA) at pH 6 for 1 h. Endogenous peroxidase was blocked using methanol and 3% H2O2 for 30 min. Primary antibodies were used for bcl-2 and for COX-2 (DAKO) at a dilution of 1:50 for 30 min. Envision (DAKO) was used as a detection system, and the immune response was visualized using diaminobenzidine (DAB) for 10 min. Adequate positive and negative control was used.

The laminas containing the tissue were observed under light microscope. The expression was semiquantitatively analysed according to the number of positive cells in a field of observation at 40X: < 1% of the cells with positive staining was considered negative (+) < 30% positive cells, (++) 30–70% positive cells, and (+++) > 70% positive cells. Similarly, the location of the immune response was examined by histological localization on: basal, suprabasal, and submucosa. The intensity of immunostaining was recorded as mild, moderate, or strong.

The statistical analysis was performed using Fisher’s exact test, and *P* < 0.05 was considered statistically significant.

## Results

The distribution of OLP and OLR groups according to age group was 59.53 SD ± 11.74 for OLP and 56.97 SD ± 15.06 for the OLR group. According to gender distribution, 79.41% (27/34) corresponded to females and 20.58% (7/34) to males for the OLR group and 84.64% (25/31) to females for the OLP group, while 19.35% (6/31) were from males.

Buccal mucosa was the most common anatomic location, occurring bilaterally in the OLP group and unilaterally in the OLR group.

The immunohistochemical analysis showed 53% (18/34) of positivity for COX-2 in the ORL group, whereas in the OLP group, 81% of the samples (25/31) were positive. The difference was statistically significant between the two groups, *P* = 0.035. The bcl-2 protein expression was 76% (26/34) in the OLR, while in the OLP group 97% (30/31) of the samples were positive. The difference was statistically significant between the two groups, *P* = 0.028.

**Figure 1.**
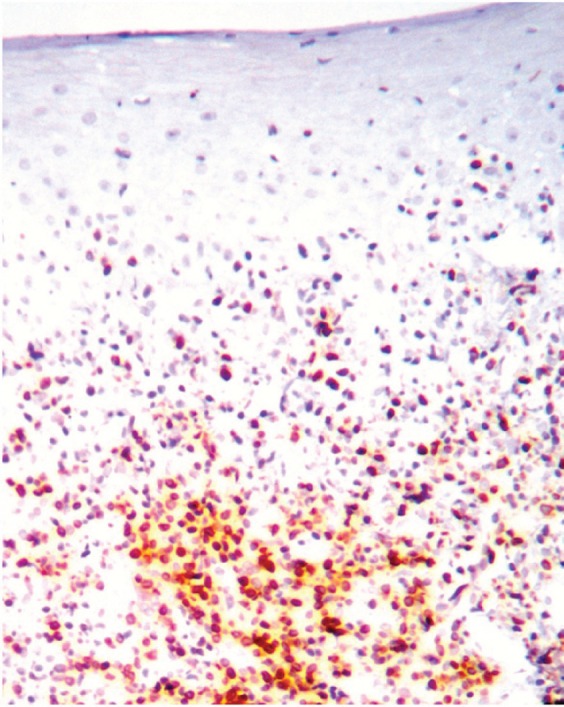
Bcl-2 expression in the lymphocytic infiltrate at basal cell layer of the epithelium.

**Figure 2.**
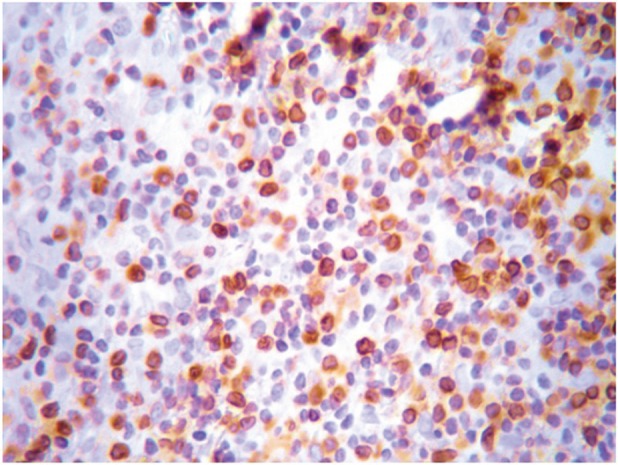
Bcl-2 expression at the lymphocytic infiltrate. Magnification 40X.

**Figure 3.**
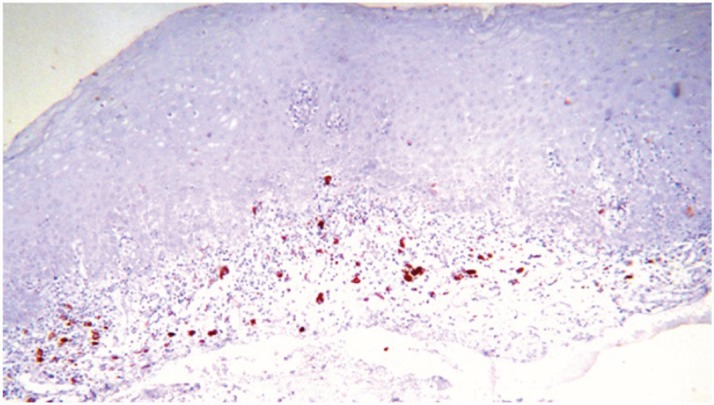
COX-2 expression at the lymphocytic infiltrate, 10X.

**Figure 4.**
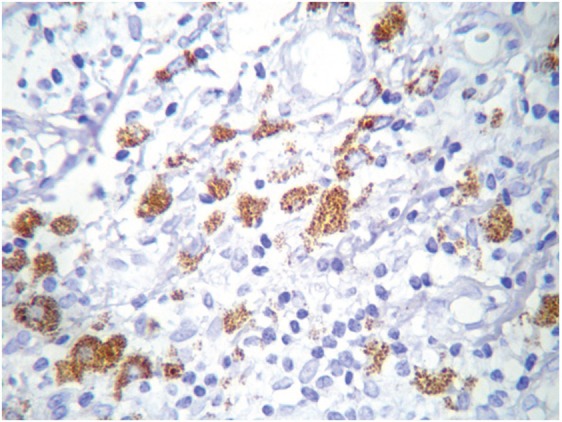
COX-2 cytoplasmic expression in macrophages at the inflammatory infiltrate. Magnification 40X.

The most frequent site of immunolabelling for bcl-2 in the OLP group was at the band-like infiltrate at the corion-epithelial junction and in the submucosa (Table 1, Figures 1 and 2). Similarly, the most frequent site of immunolabelling for COX-2 was the band-line inflammatory infiltrate in the OLR group samples. However, this marker was also observed in the basal layer and in the submucosa. (Table 1, Figures 3 and 4).

In relation to immunostaining extension, most of the OLR samples showed less than 30% extension (+). Meanwhile, (++) extension was found in four cases for bcl-2 and in three cases for COX-2. Regarding the OLP samples, the extension was mostly observed around (+) and (++) for both markers. There were no positive samples in (+++) extension for COX-2, while there were seven cases in OLP and five in OLR with (+++) extension for bcl-2 (Table 2).

Concerning the intensity of immunostaining, most cases presented a moderate intensity for both markers, but the expression of the bcl-2 protein was intense and extensive in both lesions (Table 3).

## Discussion

The age and gender most affected in this current study were women in the fifth decade of life. These data are consistent with previous reports from the literature regarding the epidemiology of OLP and OLR [1–3]. According to the anatomical location, the buccal mucosa was the most affected site in both groups as reported by the majority of authors [1–4]. Similarly, the bilateral appearance of OLP lesions was the most frequent presentation as described by Carrozo and Thrope [1].

It has been shown that bcl-2 protein plays an important role in the etiopathogenesis of some inflammatory autoimmune diseases, as well as hematopoietic malignancies, such as B cell lymphoma, in acute promyelocytic leukaemia, and non-hematopoietic malignancies, such as pancreatic beta-cell cancer [8].

The regulation of cell death is crucial in maintaining immunocompetence. In the normal inflammatory response, the immunocompetent cells are destroyed by apoptosis after the acute phase of inflammation, but in some diseases, inflammation continues on a recurrent basis [15].

The role of apoptosis in the elimination of the inflammatory cells has been observed in several inflammatory diseases, such as cutaneous leukocytoclastic vasculitis and Behçet's disease [15]. In B lymphocytes the overexpression of bcl-2 has been associated with anti-nuclear activity, such as occurs in lupus [16]. Overexpression of this oncogene appears to play a role in the malignant transformation and development of autoimmune diseases [15].

Alterations in the apoptotic mechanism during the maturation stage and lymphocytic differentiation may be related to autoimmune episodes due to a failure to eliminate the auto reactive lymphocyte clones [15]. Inappropriate peripheral apoptosis of CD4 lymphocytes causes an increased survival of the T lymphocytes and an increase in the production of autoantibodies [15].

The role of apoptosis is of critical importance in the elimination of auto reactive lymphocytes and consequently in the inhibition of autoimmunity. Lymphocytes that escape this selective process during their development can be controlled by regulatory T cells (Treg) that express the transcription factor Foxp3 (forkhead box P3), a protein called scurfin that is involved in the immune response and whose deficiency has been associated with autoimmunity [16, 17]. Cell death regulated by the intrinsic pathway of apoptosis (via bcl-2) is relevant to the normal development and function of these Treg cells. Variations in the bcl-2 family expression may influence the relative susceptibility of Treg cells to cell death, especially when exposed to stress signals of pathophysiological significance, such as those caused by the presence of glucocorticoids or the absence of certain cytokines, causing a lack of control over auto reactive lymphocytes [16, 17].

As observed in the current study results, the maintenance of a band-like lymphocytic inflammatory infiltrate across the entire corium-epithelial junction, as is observed in OLP, OLR, and other autoimmune diseases, may be associated with the overexpression of the bcl-2 protein and the capacity that it imprints on these cells to maintain an inflammatory response without undergoing apoptosis. This inflammatory response passes through the production of COX-2 and its metabolic products derived from arachidonic acid, not only in the infiltrate, but along the basal layer, and even in upper-epithelial layers. Both molecules have also been associated with genetic alterations that lead to malignancy [8, 18].

The bcl-2 protein expressed in the infiltrating lymphocytes may enable them to escape from apoptosis and prolong survival. However, it was not possible to determine from this study, whether the increase in expression of bcl-2 is an attempt to maintain the population of activated lymphocytes despite underlying etiologic factors (endogenous or exogenous) or whether it is the primary abnormality. Further studies are required to understand the biological relevance and clinical significance of lymphocytic alterations of bcl-2 in the pathogenesis of OLP and OLR.

Inflammation plays an important role in the carcinogenesis of chronic autoimmune diseases. The expression of the COX-2 enzyme is a normal event in inflammatory processes and is involved in tissue repair processes promoting angiogenesis as well as cell proliferation and differentiation. However, these effects can be detrimental when inflammation becomes chronic and persistent as occurs with OLP and OLR [18].

Inflammation is common in oral cancer. It has been associated with both the formation and the progress of oral cancer. Polymorphisms at the COX-2 promoter level may contribute to the differential expression of COX-2 and consequently to variability and susceptibility to the development of cancer. In the oral cavity, various types of lesions, including OLP, oral leukoplakia, and submucous fibrosis have malignant potential. However, the factors which influence the transformation of OLP to squamous carcinoma cells are not yet fully elucidated [7]. For Lin *et al* [19] , the COX-2-765C allele was a risk factor in oral leukoplakia which progressed to malignancy.

There is evidence indicating that COX-2 overexpression is an early event in epithelial carcinogenesis, postulating that the increased levels of COX-2 and EGFR (epidermal growth factor) in premalignant lesions constitute a mechanism for `field carcinogenesis’ [18]. In normal epithelial cells, expression of COX-2 is minimal, so that the inflammatory effect of lymphocytic infiltrate maintained by the anti-apoptotic activity of bcl-2 in OLP and OLR lesions may result in the induction of COX-2 expression, not only in the same infiltrate, but also in the adjacent epithelium. This occurrence appears to be high for both lesions, but according to the results of the current study, it is primarily expressed with OLP.

Therapeutic research with the use of specific COX-2 inhibitors to prevent malignant transformation of these lesions should prove to be an interesting topic for future studies [20].

Cortés *et al* [7] documented that COX-2 expression was higher in OLR lesions than in OLP lesions. Our study contradicts these results. This leads to a consideration of the need for a complete clinical follow up on OLP lesions of reticular appearance, although it has been suggested in several studies that erosive lesions have greater potential for malignancy [1–4].

As in the current study, Cortés *et al* [7] found increased COX-2 expression in the inflammatory infiltrate of lichenoid lesions, although it could also be observed at the epithelial level. In the current study, this epithelial pattern was most often observed in cases of OLP. These findings suggest that the aetiology and molecular pathophysiological pathways for these lesions are different.

## Conclusion

The expression of bcl-2 and COX-2 was greater in OLP lesions than in OLR lesions. The expression of bcl-2 was located primarily at the level of the band-like lymphocytic infiltrate which was observed in the corium-epithelial junction. This genetic alteration may be responsible for the maintenance of the chronic inflammatory response in both types of lesions, and with the constant expression of COX-2, may lead to the development and accumulation of oncogenic genetic alterations.

## Conflicts of interest

The study was approved by the bioethics committee of the Faculty of Dentistry at the Central University of Venezuela. All patients signed an informed consent for the biopsy procedure. The authors report no conflicts of interest with reference to the research objectives.

## Figures and Tables

**Table 1.** Immunostaining by location on both groups.

Location	bcl-2 OLP	bcl-2 OLR	COX-2 OLP	COX-2 OLR
Lymphocytic infiltrate	26	21	12	12
Basal layer	0	0	2	2
Basal/lymphocytic infiltrate	0	2	6	1
Infiltrate/submucosa	1	2	2	1
Submucosa	0	0	0	1
Lymphocytic infiltrate/basal/submucosa	0	0	0	1

**Table 2.** Immunostaining extension COX-2 and bcl-2 in both groups.

	+	++	+++
bcl-2 (OLP)	10	13	7
bcl-2 (OLR)	17	4	5
COX-2 (OLP)	19	6	0
COX-2 (OLR)	15	3	0

**Table 3.** Immunostaining of bcl-2 and COX-2 according to intensity on both groups.

	Light	Moderate	Intense
bcl-2 (OLP)	10	11	9
bcl-2 (OLR)	8	10	8
COX-2 (OLP)	7	14	4
COX-2 (OLR)	8	9	1
